# The relationship between secondhand smoke exposure in Chinese children and adolescents and renal function and hyperuricemia: a cross-sectional study

**DOI:** 10.3389/fped.2026.1793355

**Published:** 2026-06-09

**Authors:** Han Zhou, Jianxin Fu, Weiliang Liu, Changqing Liu, Yiya Liu, Meina Tian, Qianrang Zhu, Lianlong Yu, Weijie Dou

**Affiliations:** 1 Shandong Center for Disease Control and Prevention, Jinan, Shandong, China; 2 NHC Specialty Laboratory of Food Safety Risk Assessment and Standard Development (Food Microorganisms), Jinan, Shandong, China; 3 School of Food Science and Engineering, Shandong Agricultural and Engineering University, Jinan, Shandong, China; 4 Hebei Center for Disease Control and Prevention, Shijiazhuang, China; 5 Guizhou Center for Disease Control and Prevention, Guiyang, China; 6 Jiangsu Provincial Center for Disease Control and Prevention, Nanjing, China; 7 School of Public Health, Shandong Second Medical University, Weifang, Shandong, China; 8 School of Public Health, Shandong First Medical University, Jinan, Shandong, China; 9 School of Health Care Security, First Medical University & Shandong Academy of Medical Sciences Jinan, Shandong, China

**Keywords:** children and adolescents, glomerular filtration rate, hyperuricemia, mediating effect, secondhand smoke

## Abstract

**Background and objectives:**

Exposure to second—hand smoke (SHS) can potentially influence serum uric acid levels. However, there remains a relative paucity of studies on this topic among children and adolescents. This study intends to examine the association between SHS exposure and hyperuricemia (HUA) among Chinese children and adolescents, and to explore the potential mediating role of renal function in this relationship.

**Methods:**

The data for this cross-sectional study were sourced from the “Health Monitoring of Chinese Children and Lactating Women Nutrition and Health Surveillance” (CCLWNHS) carried out between 2016 and 2018. It encompassed a total of 13,062 individuals aged 6–17 from four provinces, comprising 6,536 boys and 6,526 girls. The frequency of the research subjects' exposure to SHS was acquired via a questionnaire survey. The dietary intake was collected through a 3—day 24—hour dietary retrospective questionnaire. Blood samples were gathered, and indicators such as uric acid and serum creatinine were examined. The prevalence of HUA is defined according to the fasting serum uric acid level, and the threshold differs according to age and gender. The function of the kidneys is assessed using the glomerular filtration rate (eGFR). The Chi-square test, Pearson correlation test, and logistic regression were employed to examine the associations among SHS, HUA, and eGFR. The LASSO model was utilized to select variables, and the XGBoost model was employed to forecast the occurrence of HUA. The mediation effect and Sobel test were utilized to analyze the effect of eGFR on SHS and HUA.

**Result:**

The prevalence rate of HUA among children and adolescents aged 6–17 stands at 20.5%. The exposure rate of SHS in the study population was found to be 41%. When compared with daily exposure to SHS, no exposure to SHS emerged as a protective factor for HUA (OR = 0.811, 95% CI: 0.698, 0.943). The results of the stratified analysis indicated that, in comparison to boys exposed to SHS on a daily basis, boys not exposed to SHS were identified as a protective factor for HUA (OR = 0.747, 95% CI: 0.613, 0.911). Mediation analysis demonstrated that eGFR mediated 10.42% of the association between SHS and HUA in boys. Furthermore, the mediating effect of eGFR on the relationship between SHS exposure and HUA in boys aged 12–17 reached 100%.

**Conclusion:**

Exposure of children and adolescents, particularly boys, to SHS may elevate the incidence of HUA by impairing the glomerular filtration function. Therefore, it is imperative to enforce the smoking ban in public places more stringently.

## 1 Introduction

Uric acid serves as the ultimate product of purine metabolism within the human body. The pathogenesis of hyperuricemia (HUA) encompasses excessive uric acid production and diminished uric acid excretion by the kidneys (1). An abnormally elevated serum uric acid level not only instigates gout attacks and kidney diseases but also escalates the risks of cardiovascular diseases, hypertension, dyslipidemia, diabetes, and even death (2, 3), thereby imposing heavier burdens on society and families. Nevertheless, in contrast to adults, the incidence and severity of HUA in children and adolescents are seriously under—recognized. The Clinical Practice Consensus on the Management of HUA and Gout in Chinese Adolescents, published in 2025, suggests that the prevalence of HUA among Chinese adolescents lies between 26.6% and 42.3% (4). Another pooled analysis involving 54,580 participants shows that the overall prevalence of HUA among children and adolescents aged 3–19 in China is estimated to be 23.3% (5). The risks of kidney and cardiovascular complications and death in the adolescent HUA population are higher (6), and the safety verification of relevant treatment drugs (such as allopurinol) for children and adolescents has not been established. However, research has indicated that serum uric acid levels are also affected by lifestyle, genetic, and dietary factors.

Second hand smoke (SHS) refers to the exposure of non—smokers to tobacco smoke in the environment. It represents an unhealthy lifestyle. In 1992, the National Cancer Research Institute classified SHS as a first—class carcinogen (7). Studies have demonstrated that both active and passive smoking among adults can result in an elevation of uric acid levels (8, 9), and smokers are also susceptible to acute or chronic kidney damage. Nevertheless, certain metabolomics studies have indicated that nicotine can directly expedite the decomposition rate of purines, and this might be unrelated to kidney damage (10, 11). The relationship between SHS and HUA and the role of renal function in this regard remain ambiguous. Moreover, there are still relatively few studies on the association between children and adolescents' exposure to SHS and uric acid. Globally, 40% of children and adolescents are exposed to SHS (12). According to the report of the World Health Organization (WHO), 180 million children in China are exposed to SHS (13). Compared with adults, it is easier to modify the behavior and lifestyle of children, and the long—term benefits achieved are more substantial.

Therefore, we conducted a cross-sectional study among children and adolescents in China. The objective was to explore the relationship between exposure to SHS and blood uric acid levels, and to evaluate the potential mediating role of renal function in this association.

## 2 Materials and methods

### 2.1 Study population

The data are derived from the Health Monitoring of the China Children and Lactating Women Nutrition and Health Surveillance (CCLWNHS) spanning from 2016 to 2018. This survey, a national—scale one, encompasses all 31 provinces and is conducted by the Chinese Center for Disease Control and Prevention. The health monitoring of children and lactating mothers in China employs a multi—stage stratified random sampling approach. While guaranteeing both national and provincial representativeness of the monitoring samples, it also takes into account four types of regions: large cities, medium and small cities, ordinary rural areas, and poor rural areas. Ultimately, 150 monitoring sites (districts and counties) across 31 provinces (autonomous regions and municipalities) in the country were selected. Within each monitoring point, five schools (comprising two primary schools, two junior high schools, and one senior high school) were chosen, and two classes were randomly picked from each school. Twenty—eight students were randomly selected from each class (for detailed sampling steps, refer to Supplementary Appendix S1). Given that the study involved minors, the opinions of their families were initially solicited. After obtaining the consent of family members, schoolteachers, and the minors themselves, an informed consent form was officially signed (Supplementary Appendix S2). After acquiring informed consent, the research subjects completed a comprehensive questionnaire that covered basic information, living habits, dietary surveys, physical examinations, and laboratory tests such as fasting venous blood collection and random urine analysis. (Ethical review was approved by the Ethical Review Committee of the Chinese Center for Disease Control and Prevention, number: 201614. See Supplementary Appendix S3).

**Figure 1:**
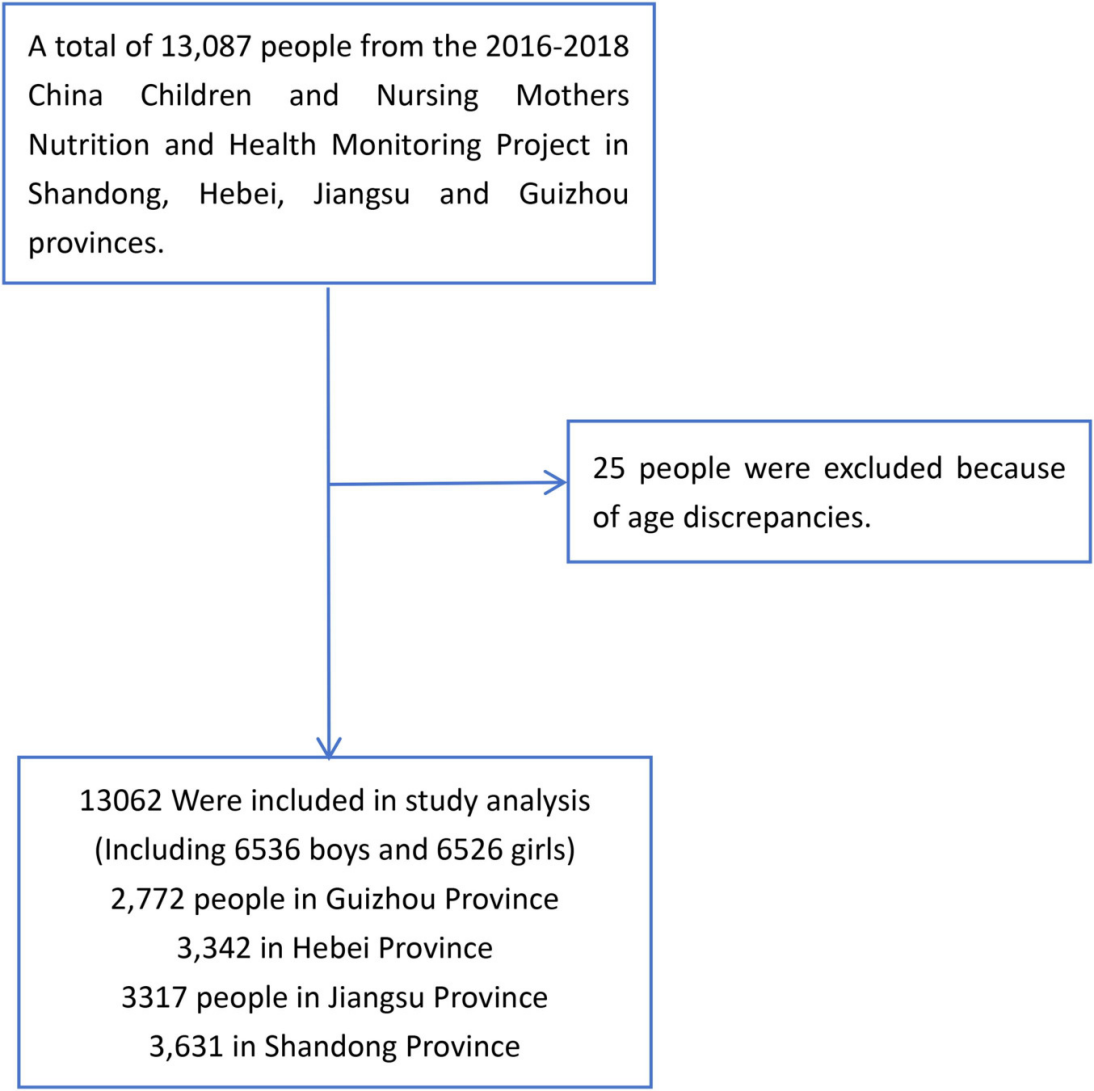
Study population.

Among them, the height was measured using the TZG altimeter, which had a minimum scale of 0.1 cm. The weight was measured using a unified electronic scale with a minimum scale of 0.1 kg. Blood pressure was measured using the Omron HBP1300 electronic blood pressure monitor, which had an accuracy of 1 mmHg and was equipped with a children's cuff. As part of the monitoring project, this study included the monitoring data from four provinces, namely Shandong Province, Guizhou Province, Hebei Province, and Jiangsu Province, involving a total of 13,062 participants, including 6,536 males and 6,526 females. The specific details of the data are shown in Figure 1.

### 2.2 Inclusion and exclusion criteria

This study incorporated children and adolescents aged 6–17 who furnished comprehensive questionnaire information, underwent medical physical examinations, and provided laboratory test data. Participants who failed to meet the age criteria, had incomplete questionnaires, or had insufficient data on physical measurements and blood indicators were excluded.

### 2.3 Blood indicators

Fasting venous blood was collected from children and adolescents aged 6–18 at all monitoring sites, with a total volume of 6 mL (including 2 mL of anticoagulated blood and 4 mL of non—anticoagulated blood). Routine biochemical indicators are centrally tested by provincial disease control centers. Among these indicators, blood glucose is detected using the hexokinase method, blood uric acid using the uricase method, cholesterol using the cholesterol oxidase method, serum creatinine using the enzymatic method, and triglycerides using the free glycerol method. All tests are conducted in strict accordance with the quality control and operation requirements of national project laboratories.

### 2.4 Research variables and variable definitions

During the survey, children and adolescents aged 6–17 were categorized as day students and boarders. The dietary intake of the research participants was investigated using a 3—day 24—hour dietary retrospective questionnaire (14). Specifically, a continuous three—day food intake survey was conducted for all family members of the day students. This involved weighing the cooking oil and seasonings used in the family for three days and recording the number of family meals. For boarding students, a three—day weighing of cooking oil and seasonings in the school cafeteria was carried out, along with the registration of the number of diners in the cafeteria and a dietary review. Physical aids such as maps and models were utilized to facilitate the completion of the questionnaire. The personal daily energy intake data were obtained from the aforementioned dietary retrospective questionnaire. Passive smoking is defined as exposure to SHS (15), where SHS refers to the smoke exhaled by smokers and the smoke emitted from the ends of cigarettes during smoking. BMI is calculated by dividing weight (in kilograms) by the square of height (in meters).

The diagnostic criteria for HUA in adolescents (16, 17) are as follows: serum uric acid level >500 μmol/L at 1–12 months, >320 μmol/L at 1–10 years old; for 11–15—year—old males, >470 μmol/L, and for 11–15—year—old females, >350 μmol/L; for those 15 years old and above, the same adult standard applies (both males and females >420 μmol/L).

All laboratory tests were conducted in laboratories that hold the ISO15189 medical laboratory accreditation certificate and the American CAP laboratory accreditation certificate. Among them, serum creatinine was measured using the enzymatic method, with the formulation being liquid dual reagent, and the linear range was 5–2,700 μmol/L. The glomerular filtration rate (GFR) reflects the efficiency of the glomerulus in filtering blood and removing waste and is often used to indicate changes in renal function. Estimated GFR (eGFR) is calculated using the Schwartz formula from height and serum creatinine. Forty—three different coefficients will be used for different populations, including a K of 0.55 for participants aged 6–12, 0.77 for men over 12 years old, and 0.55 for women (18, 19).

$$\mathrm{eGFR}[\mathrm{ml}/(\min\;1.73\;m^{2}]=k\times \mathrm{height}\times 88.4/\mathrm{Serum}\;\mathrm{creatinine}$$

### 2.5 Statistical analysis

The measurement data were presented as mean ± standard deviation (mean ± SD), while count data were reported as frequency. Q − Q plots and P − P plots were used to verify the normal distribution of the data. Since the sampling design of this study is rigorous, we assume that the missing values are random. The PMM (Predictive Mean Matching) method in multiple imputation is adopted to handle the missing values, thus better preserving the natural variability of the data. The *t*-test was employed to compare the rates between two groups, and the chi—square test was used to compare the rates of multiple groups. Binary logistic regression was utilized to analyze the relationship between SHS and HUA in people of different ages and genders. The Variance Inflation Factor (VIF) is used to assess the problem of multicollinearity in linear regression. When there is multiple collinearity in the intake of dietary nutrients, the Chinese Residents' Dietary Protein Reference Intake Percentage is used for correction (20). Among them, all the dietary nutrient indicators were standardized based on body weight. The original BMI of each subject was subjected to Z-score correction.

**Figure 2:**
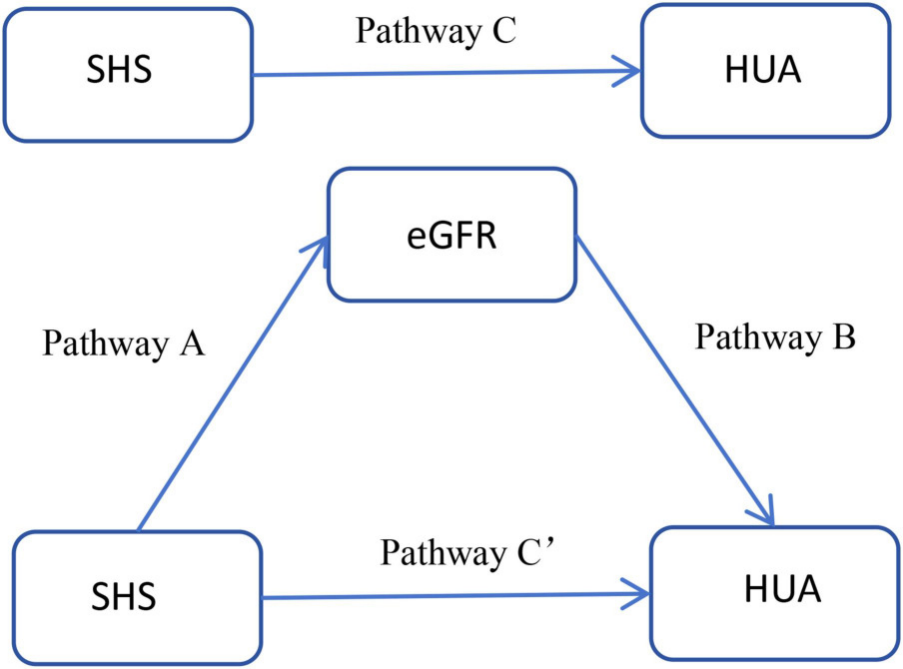
Model of the potential mediating effect of eGFR on the relationship between SHS of children and adolescents aged 6-17 years and HUA.

There is a correlation between eGFR and blood uric acid levels (21). Therefore, we investigated whether eGFR also plays a certain role in the process where SHS intake affects HUA. Based on the theory of mediating effects proposed by Baron and Kenny (Causal stepwise regression test) (22, 23), eGFR was considered as a mediator factor (M), SHS (X) was considered as an independent variable, and HUA was considered as a dependent variable (Y) for mediating effect analysis. The total effect (c) of factor X on factor Y can be decomposed into direct effect (c′) and mediating effect (ab). “a” was the effect of factor X on factor M, and “b” was the effect of factor M on factor Y after adjusting for factor X (Figure 2). The specific steps are as follows: First, analyze the regression of X on Y and test the significance of regression coefficient c. Second, analyze the regression from X to M and test the significance of the regression coefficient a. Third, analyze the regression from X to Y with the addition of the mediating variable M, and test the significance of the regression coefficients b and c′. Among them, when both a and b are significant, the significance of c′ is tested to judge the mediation effect; when at least one of a and b is not significant, the Sobel test is used to determine whether a mediation effect is present. Furthermore, the total effect of the mediating effect is c, and the indirect effect is a*b = c—c′. From the equation of total effect = direct effect + indirect effect, it can be concluded that the proportion of the mediating effect = indirect effect/total effect (%). The confidence interval of the indirect effect was calculated using the Bootstrap method. If the interval does not include 0, it is considered that the mediating effect is statistically significant. All statistical tests were two—sided at *α* = 0.05, and all analyses were conducted using SPSS 25.0.

To reduce the impact of multicollinearity on the regression results, all selected covariates were screened using the least absolute shrinkage and selection operator (LASSO). The analysis of LASSO regression involves two steps: first, using the trajectory plot to determine the optimal K value; second, inputting the K value to conduct regression modeling. In addition to addressing the issue of collinearity among variables, it can also perform “feature selection”, that is, identify the meaningful independent variables X (features). A LASSO model (24) was developed in RStudio using the CRAN package keras (25, 26).

The XGBoost (extreme gradient boosting) model based on GBDT (gradient boosting decision tree) is used to predict the occurrence of HUA, and the SHAP values are utilized to explain the XGBoost model (27). This part of the content was completed using Python 3.6 version, Xgboost 0.82 version, and shap 0.28.5 version.

## Results

**Figure 3:**
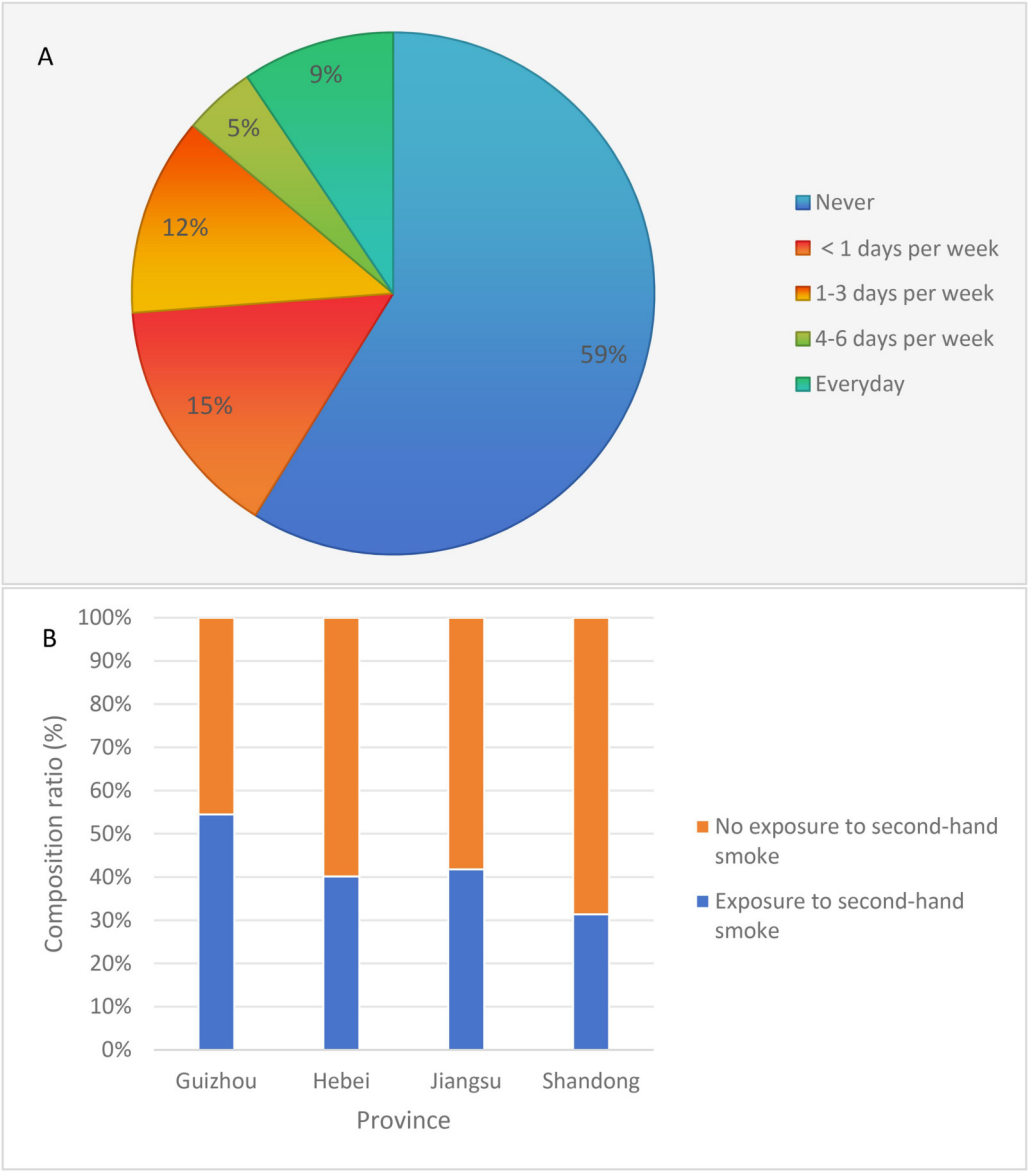
The **A** represents a pie chart, which shows the frequency distribution of people's exposure to SHS in the population. Among them, 41% of the people have been exposed to SHS. Panel **B** is a stacked bar chart showing the percentage of people exposed to SHS in different provinces. It compares the distribution of this indicator (whether exposed to SHS) across different provinces.

After excluding 25 participants whose ages did not meet the requirements, a total of 13,062 individuals (comprising 6,536 boys and 6,526 girls) were included in this study (see Figure 1). Table 1 indicates that a total of 2,683 children and adolescents were diagnosed with HUA, with a prevalence rate of 20.5%. Among them, the prevalence rates were 22.5% in Guizhou Province, 16.6% in Hebei Province, 21.6% in Jiangsu Province, and 21.7% in Shandong Province. The HUA patient group is more likely to consist of frequent SHS exposed individuals, heavy drinkers, snorers, those with a high BMI, and individuals with elevated total cholesterol and triglycerides. Figure 3 demonstrates that the proportion of people exposed to SHS in the total population is 41%. There are variations in the frequency of exposure to SHS across different provinces.

**Table 1:** Basic characteristics of children and adolescents aged 6–17 years in four provinces of China.

Characteristic	ALL		Normal		HUA		*χ*^2^/t	*P*
	*N*	(Weighted, %)	*N*	(Weighted, %)	*N*	(Weighted, %)		
No. of participants	13,062	100	10,379	79.5	2,683	20.5		
Girls	6,536	50.0	5,344	81.8	1,192	18.2	42.513	0.000
Age							0.021	0.885
6–11 years	8,472	64.9	6,735	79.5	1,737	20.5		
12–17 years	4,590	35.1	3,644	79.4	946	20.6		
Province where participants located								
Guizhou	2,772	21.2	2,148	77.5	624	22.5	43.489	0.000
Hebei	3,342	25.6	2,787	83.4	555	16.6		
Jiangsu	3,317	25.4	2,600	78.4	717	21.6		
Shandong	3,631	27.8	2,844	78.3	787	21.7		
Exposure to second-hand smoke							17.621	0.001
Never	7,686	58.8	6,161	80.2	1,525	19.8		
<1 days per week	1,956	15.0	1,584	81.0	372	19.0		
1–3 days per week	1,606	12.3	1,235	76.9	371	23.1		
4–6 days per week	582	4.5	450	77.3	132	22.7		
Everyday	1,232	69.0	949	77.0	283	23.0		
Drink							17.891	0.000
Yes, within 30 days	476	3.6	346	72.7	130	27.3		
Yeah, 30 days ago	930	7.1	719	77.3	211	22.7		
Never	11,629	89.0	9,291	79.9	2,338	20.1		
Not clear	27	0.2	23	85.2	4	14.8		
Take care of your daily life							4.304	0.636
Mother	10,102	77.5	8,059	79.8	2,043	20.2		
Father	745	5.7	577	77.4	168	22.6		
Grandparents/maternal grandparents	1,953	15.0	1,533	78.5	420	21.5		
Siblings of children	39	0.3	30	76.9	9	23.1		
Other relatives	128	1.0	103	80.5	25	19.5		
The nurse	8	0.1	7	87.5	1	12.5		
Other	57	0.4	44	77.2	13	22.8		
Unable to fall asleep within 30 min of going to bed			2.871	0.238				
Most of the time	1,192	9.1	937	78.6	255	21.4		
Occasionally	5,150	39.5	4,129	80.2	1,021	19.8		
No	6,693	51.3	5,290	79.0	1,403	21.0		
Snoring							19.712	0.000
Most of the time	300	2.3	213	71.0	87	29.0		
Occasionally	2,870	22.0	2,238	78.0	632	22.0		
No	9,864	75.7	7,904	80.1	1,960	19.9		
You wake up thinking you didn't sleep well					2.561	0.278		
Most of the time	1,785	13.7	1,395	78.2	390	21.8		
Occasionally	5,184	39.8	4,115	79.4	1,069	20.6		
No	6,065	46.4	4,845	79.9	1,220	20.1		
Take part in sports on campus							1.322	0.970
Everyday	3,153	30.7	2,507	79.5	646	20.5		
4–6 days/week	2,902	28.2	2,301	79.3	601	20.7		
2–3 days/week	3,131	30.5	2,470	78.9	661	21.1		
1 days/week	840	8.2	667	79.4	173	20.6		
1 day/month	49	0.5	39	79.6	10	20.4		
<1 day/month	46	0.4	37	80.4	9	19.6		
Never	153	1.5	126	82.4	27	17.6		
BMI SDS[M(P25,P75)]	−0.19	(−0.74,0.52)	−0.26	(−0.76,0.40)	0.13	(−0.58,1.03)	433.798	0.000
Uric acid[mean(SE)], μmol/L	313.76	83.09	287.57	61.91	414.25	77.24	7,992.403	0.000
Fasting blood glucose[mean(SE)], mmol/L	5.06	0.58	5.06	0.57	5.06	0.60	0.023	0.881
Total cholesterol[mean(SE)], mmol/L	3.87	0.72	3.84	0.71	3.95	0.74	45.497	0.000
Triglyceride[mean(SE)], mmol/L	0.91	0.41	0.89	0.38	0.10	0.52	143.240	0.000
Systolic blood pressure[mean(SE)], mmHg	113.04	12.02	112.39	11.68	115.56	12.94	147.518	0.000
Diastolic blood pressure[mean(SE)], mmHg	67.33	8.92	67.23	8.89	67.69	9.03	5,530	0.019
eGFR[mean(SE)], mL/(min 1.73 m^2^)	152.97	27.09	154.46	27.61	147.25	24.15	152.502	0.000
Standardized energy intake[mean(SE)], kcal/kg/d	58.81	44.01	59.75	45.15	55.14	39.08	23.202	0.000
Protein RNI percentage correction[mean(SE)], %	2.42	1.93	2.42	1.97	2.43	1.80	0.043	0.835
Standardized fat intake[mean(SE)], g/kg/d	1.20	1.46	1.23	1.53	1.10	1.15	17.697	0.000
Standardized carbohydrates intake [mean(SE)], g/kg/d	9.93	7.29	10.06	7.46	9.40	6.60	17.534	0.000

Based on the results of variable screening (the relevant results can be found in Supplementary Appendix S4), we carried out a further stratified analysis. Table 2 depicts the relationship between SHS exposure and HUA in different age and gender groups. Binary logistic regression was performed with daily exposure to SHS as the reference. The results demonstrated that non—exposure to SHS was a protective factor for HUA compared to daily exposure to SHS [odds ratio [OR] = 0.864, 95% confidence interval [CI]: 0.772, 0.966]. After adjusting for factors such as age, sex, body mass index (BMI), inability to fall asleep within 30 min of going to bed, snoring, waking up feeling that one didn't sleep well, total cholesterol, triglyceride, energy, and protein, the results remained stable (OR = 0.811, 95% CI: 0.698, 0.943). The stratified analysis results indicated that, compared with boys exposed to SHS daily, non—exposed boys had a protective factor for HUA (OR = 0.742, 95% CI: 0.614, 0.896). For children and adolescents aged 12–17, non—exposure to SHS was a protective factor for HUA (OR = 0.647, 95% CI: 0.507, 0.824) (compared with daily exposure to SHS). The results for “girls” (OR = 1.000, 95% CI: 0.797, 1.225) and the “6–11 years old age group” (OR = 0.943, 95% CI: 0.788, 1.129) were not statistically significant. The results remained stable after adjusting for age, gender, and BMI (boys: OR = 0.729, 95% CI: 0.600, 0.885; for ages 12–17: OR = 0.768, 95% CI: 0.594, 0.992). After further adjusting for sleep factors, total cholesterol, triglycerides, energy, and protein, exposure to SHS still remained a risk factor for HUA in girls (OR = 0.747, 95% CI: 0.613, 0.911).

**Table 2:** Association between frequency of SHS exposure and HUA in different sexes and age.

		B	S.E.	wald	*p*	OR	95%CI	OR(95%CI)		
								Model 1	Model 2	Model 3
Total	Exposure to second-hand smoke^a^									
	Never	−0.146	0.057	6.605	**0** **.** **010**	**0**.**864**	**(0.772, 0.966)**	**0.851** (**0.763, 0.949)**	**0.812** (**0.699, 0.944)**	**0.811** (**0.698, 0.943)**
	<1 days per week	−0.189	0.070	7.728	**0**.**007**	**0**.**828**	**(0.722, 0.950)**	**0.838** (**0.732, 0.960)**	**0.797** (**0.665, 0.955)**	**0.797** (**0.665, 0.955)**
	1–3 days per week	0.010	0.069	0.019	0.889	1.101	(0.881, 1.157)	1.002 (0.877, 1.144)	1.016 (0.846, 1.221)	1.015 (0.845, 1.220)
	4–6 days per week	−0.013	0.093	0.019	0.891	0.987	(0.823, 1.184)	0.987 (0.826, 1.179)	1.016 (0.796, 1.297)	1.014 (0.794, 1.294)
Girls	Exposure to second-hand smoke^a^									
	Never	0.000	0.116	0.000	0.999	1.000	(0.797, 1.255)	1.023 (0.811, 1.290)	0.994 (0.786, 1.258)	0.997 (0.788, 1.261)
	<1 days per week	−0.135	0.139	0.956	0.328	0.873	(0.666, 1.146)	0.943 (0.714, 1.246)	0.925 (0.698, 1.226)	0.927 (0.699, 1.228)
	1–3 days per week	0.153	0.141	1.177	0.278	1.166	(0.884, 1.537)	1.207 (0.909, 1.604)	1.194 (0.896, 1.590)	1.197 (0.898, 1.594)
	4–6 days per week	0.059	0.198	0.090	0.764	1.061	(0.720, 1.565)	1.081 (0.726, 1.611)	1.073 (0.716, 1.607)	1.063 (0.709, 1.593)
Boys	Exposure to second-hand smoke^a^									
	Never	−0.299	0.096	9.645	**0**.**002**	**0**.**742**	**(0.614, 0.896)**	**0.729** (**0.600, 0.885)**	**0.747** (**0.612, 0.911)**	**0.747** (**0.613, 0.911)**
	<1 days per week	−0.286	0.117	5.972	**0**.**015**	**0**.**751**	**(0.597, 0.945)**	**0.756** (**0.597, 0.957)**	**0.761** (**0.599, 0.968)**	**0.762** (**0.599, 0.968)**
	1–3 days per week	−0.081	0.118	0.463	0.496	0.923	(0.732, 1.163)	0.921 (0.726, 1.169)	0.936 (0.734, 1.193)	0.936 (0.735, 1.193)
	4–6 days per week	−0.102	0.152	0.454	0.501	0.903	(0.671, 1.215)	0.942 (0.694, 1.278)	0.982 (0.721, 1.339)	0.983 (0.721, 1.340)
6–11 years	Exposure to second-hand smoke^a^									
	Never	−0.058	0.092	0.406	0.524	0.943	(0.788, 1.129)	0.905 (0.754, 1.088)	0.893 (0.742, 1.076)	0.877 (0.729, 1.055)
	<1 days per week	−0.159	0.115	1.905	0.168	0.853	(0.681, 1.069)	0.843 (0.671, 1.061)	0.840 (0.666, 1.059)	0.841 (0.667, 1.059)
	1–3 days per week	0.060	0.115	0.268	0.605	1.061	(0.847, 1.330)	1.059 (0.842, 1.333)	1.055 (0.836, 1.331)	1.038 (0.824, 1.309)
	4–6 days per week	−0.033	0.152	0.048	0.826	0.967	(0.719, 1.302)	0.988 (0.729, 1.338)	1.004 (0.738, 1.364)	0.990 (0.729, 1.031)
12–17 years	Exposure to second-hand smoke^a^									
	Never	−0.436	0.124	12.415	**0**.**000**	**0**.**647**	**(0.507, 0.824)**	**0.768** (**0.594, 0.992)**	0.800 (0.614, 1.042)	0.799 (0.614, 1.041)
	<1 days per week	−0.399	0.142	7.931	**0**.**005**	**0**.**671**	**(0.508, 0.886)**	0.780 (0.583, 1.045)	0.787 (0.584, 1.061)	0.787 (0.584, 1.061)
	1–3 days per week	−0.094	0.146	0.410	0.522	0.910	(0.683, 1.213)	1.005 (0.743, 1.360)	1.045 (0.767, 1.423)	1.048 (0.769, 1.427)
	4–6 days per week	0.005	0.197	0.001	0.978	1.005	(0.683, 1.479)	1.014 (0.677, 1.521)	1.062 (0.702, 1.607)	1.057 (0.698, 1.599)

a Daily exposure to SHS was used as the reference level.

Model 1was adjusted for sex (boys or girls). age (years), BMI (kg/m^2^). Model 2 additionally adjusted Unable to fall asleep within 30 min of going to bed (Most of the time, Occasionally, No), Snoring (Most of the time, Occasionally, No), You wake up thinking you didn't sleep well (Most of the time, Occasionally, No), Total cholesterol, Triglyceride and Standardized energy intake. Model 3 additionally adjusted Protein RNI percentage correction.

The values in bold indicate statistical significance.

**Figure 4:**
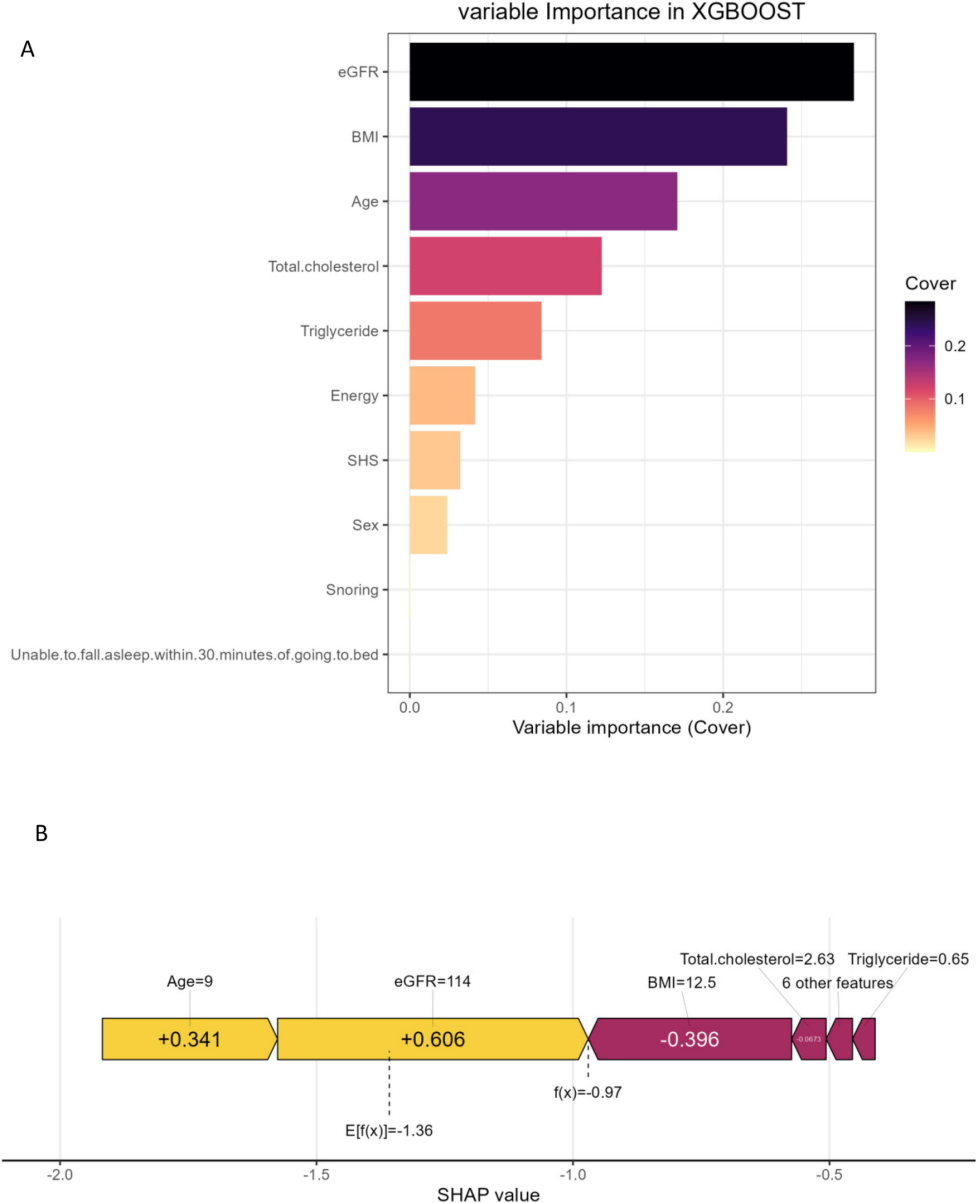
**A** represents the variable importance analysis output by XGBOOST. In this analysis, the variables that are more important for HUA have longer graphs and darker colors. **B** represents the explanation for the prediction of a single sample. The prediction starts from the baseline, which is the constant of the explanatory model. Each attribution value is an arrow that either increases (positive value) or decreases (negative value) the prediction.

**Figure 5:**
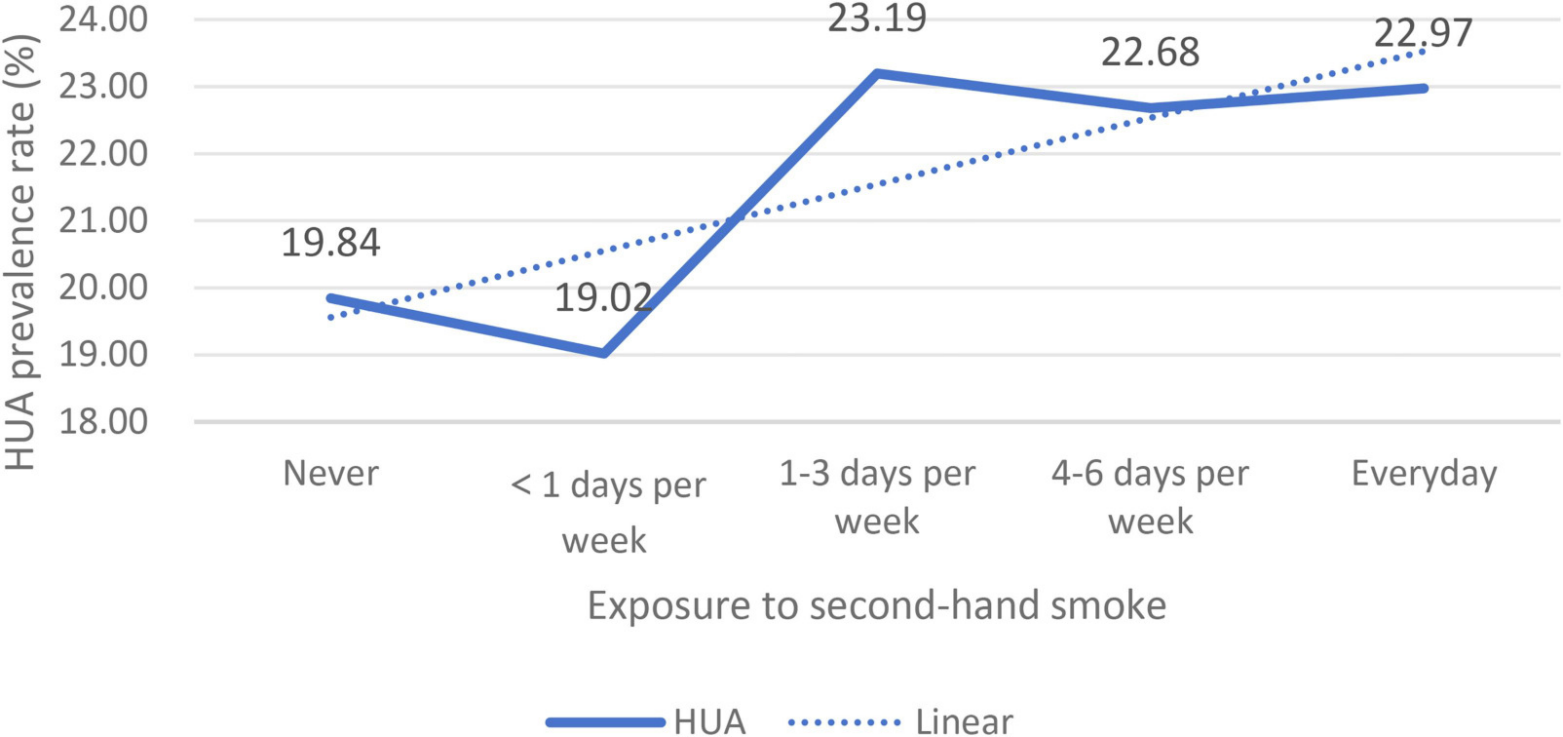
The prevalence of HUA is correlated with exposure to SHS.

The analysis results of the XGBoost model (Figure 4) show that eGFR makes the greatest contribution to predicting the HUA outcome and is positively correlated with it. There is also a correlation between HUA prevalence and exposure to SHS, with a Pearson correlation coefficient of 0.030 and *P* = 0.001 (Figure 5).

**Figure 6:**
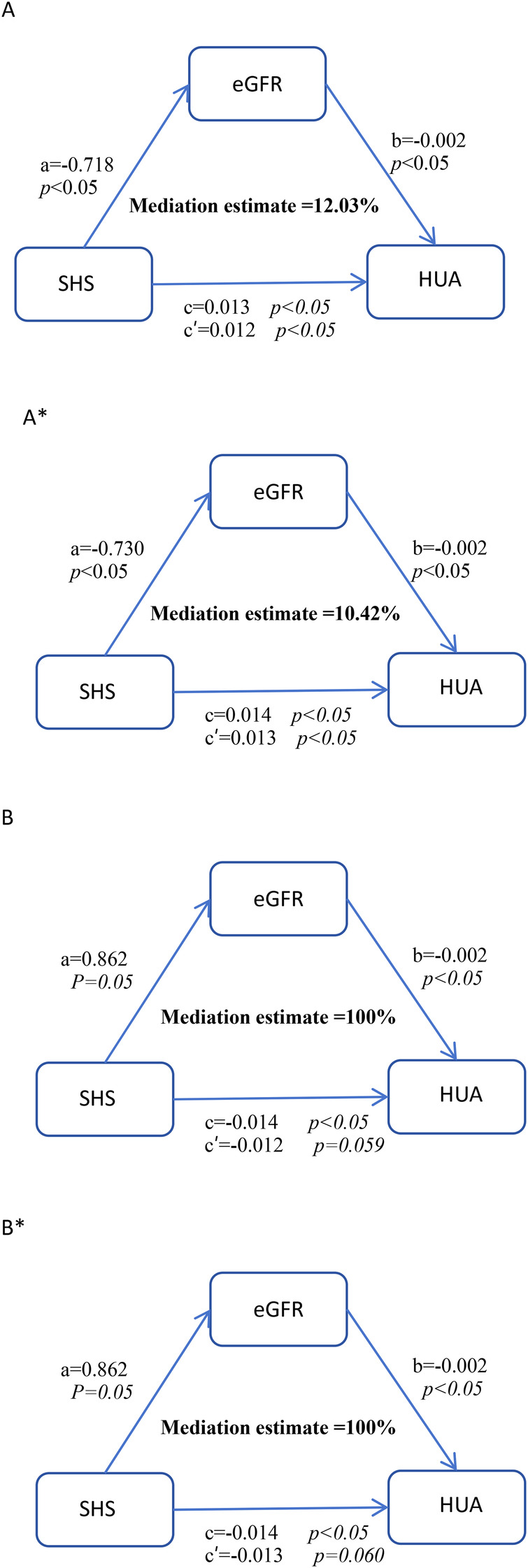
Mediating effects of eGFR on the associations of SHS with HUA. **A** represents the mediating effect among boys, and **B** represents the mediating effect among boys aged 12–17. The models were adjusted for sex (boys or girls), age (years), BMI (kg/m^2^), Unable to fall asleep within 30 min of going to bed (Most of the time, Occasionally, No), Snoring (Most of the time, Occasionally, No), You wake up thinking you didn't sleep well (Most of the time, Occasionally, No), Total cholesterol, Triglyceride and Standardized energy intake. * Indicates an addition of Protein RNI percentage correction correction based on A and B.

We employed a causal stepwise regression test to model the mediating effect of eGFR on the association between SHS exposure and HUA. Our findings indicated that eGFR was significantly associated with SHS (*p* < 0.05) and HUA (*p* < 0.05). Mediation analyses demonstrated that eGFR mediated 10.42% of the association between SHS and HUA among boys. Moreover, in boys aged 12–17, the mediating effect of eGFR on the relationship between SHS exposure and HUA reached 100% (see Figure 6). All these results were adjusted for sex (boys or girls), age (years), body mass index (BMI, kg/m^2^), the ability to fall asleep within 30 min of going to bed (most of the time, occasionally, no), snoring (most of the time, occasionally, no), the perception of poor sleep upon waking up (most of the time, occasionally, no), total cholesterol, triglyceride, energy, and protein.

## 4 Discussion

This study reveals that the overall prevalence of HUA among children and adolescents in four provinces of China is 20.5%, and 41% of these children and adolescents are exposed to SHS. The prevalence of HUA exhibits substantial variation among children and adolescents. In certain regions, the prevalence is less than 10%, whereas in others, it surpasses 30%. This variation can be attributed to disparities in the age groups of the participating populations, gender, HUA diagnostic criteria, and ethnicities across different studies (28). The results of this study do not show a significant difference from those previously obtained using the same database in terms of the prevalence of HUA (22.8%) (29). Research results regarding the probability of children and adolescents being exposed to SHS in their environment exhibit significant variations both within the country and globally. Among these results, the probability range of 11–19—year—old school—going adolescents in Colombia being exposed to SHS lies between 19.6% and 42.1% (30). In Lagos, Nigeria, 7.6% of teenagers aged 12.9 ± 1.43 years frequently come into contact with SHS (31). In China (32), the probability of having household SHS is 41.7%. A survey encompassing 122 countries and involving 557,332 adolescents aged 11–17 revealed that 35% of these teenagers had been exposed to SHS at home, while 46.4% had been exposed to SHS at school (33). Although these data, which encompass our research results, cannot fully represent the actual prevalence of SHS, they do underscore the substantial number of teenagers exposed to SHS.

Tobacco has a long—term impact on the health of young people. Our research shows that exposure to SHS is a risk factor for HUA. SHS (34), also referred to as passive smoking or environmental tobacco smoke, consists of sidestream smoke generated by the burning of tobacco products like cigarettes, cigars, or pipes, along with mainstream smoke exhaled by smokers. Exposure to SHS has been categorized as a “first-class” carcinogen (a known human carcinogen) by the International Agency for Research on Cancer. Numerous studies (35, 36) have shown that SHS has numerous adverse effects on the health of both adults and children, including hazards to the respiratory system, acute and chronic cardiovascular effects, and lung cancer. Previous studies (37, 38) have shown that smoking is a risk factor for the progression of chronic kidney disease, and exposure to SHS can significantly increase the level of uric acid (39). According to the results of this study, exposure to SHS serves as a risk factor for HUA in children and adolescents. Several studies have indicated that harmful substances in SHS, like cotinine, exert a threshold effect on HUA (40). The concentration of cotinine in the serum can accurately reflect the extent to which the human body has been exposed to tobacco smoke (41). All the aforementioned evidence indicates that exposure to SHS plays a significant role in the development of kidney diseases.

The eGFR serves as the gold—standard for assessing kidney filtration (42). Simultaneously, HUA is intricately linked to kidney diseases (43). This study reveals that eGFR plays a mediating role in HUA induced by SHS, particularly among boys aged 12–17. This finding suggests that the health risks imposed by SHS on children and adolescents are comprehensive and not confined to the influence of a single indicator. A study (44) has demonstrated that passive smoking can modify the circulating naïve/memory lymphocyte T-cell subpopulations across a child's body. This also suggests that the harm inflicted by smoking on the body is not restricted to a specific area. Previous studies have shown (45) that girls exposed to SHS exhibit a higher allergic reaction than boys. In a study conducted in Malaysia (46), it was found that exposure to second-hand smoke among boys was associated with tooth decay. A study from an expectant pregnancy and childbirth cohort showed that when mothers and children were exposed to SHS before birth and during early childhood, the food reactivity results for girls and boys were opposite (47). This finding, to some extent, suggests that there are gender differences in the impact of SHS on children and adolescents. The prevalence of SHS exposure and the serum cotinine levels resulting from SHS exposure among adolescents both increase with age (12, 48). Moreover, the harmful effects of toxic substances in tobacco on the body also become more pronounced. This could be the reason why the mediating effect of eGFR is more significant in the 12–17 age group.

There are several limitations in our study that merit attention. First, in this study, the assessment of exposure to SHS only considered the frequency and overlooked the duration factor. This oversight may result in specific errors when interpreting the results. Second, despite the inclusion of data from four provinces nationwide, the study may not comprehensively represent the SHS exposure and HUA levels of children and adolescents across China. Subsequently, based on the location of SHS exposure, the duration of exposure, and parents' attitudes towards smoking prohibition, further research can be carried out on measures to prevent children and teenagers from being exposed to SHS or on methods to mitigate the harm of SHS to the body.

## 5 Conclusion

In conclusion, the probability of children and adolescents being exposed to SHS remains high. Exposure of children and adolescents to SHS may elevate the incidence of HUA by impairing the glomerular filtration function, particularly in boys. It is imperative to more strictly enforce the smoking ban in public places. Moreover, special attention ought to be given to the harm of SHS in households to the physical and mental development of children and adolescents.

## Data Availability

The data supporting the findings of this study are available from the corresponding author upon reasonable request.
