# Supplementary figures and images for: The relationship between secondhand smoke exposure in Chinese children and adolescents and renal function and hyperuricemia: a cross-sectional study
Source: Front Pediatr. 2026 Jun 9;14:1793355. doi: 10.3389/fped.2026.1793355 (PMC13287010; doi:10.3389/fped.2026.1793355)

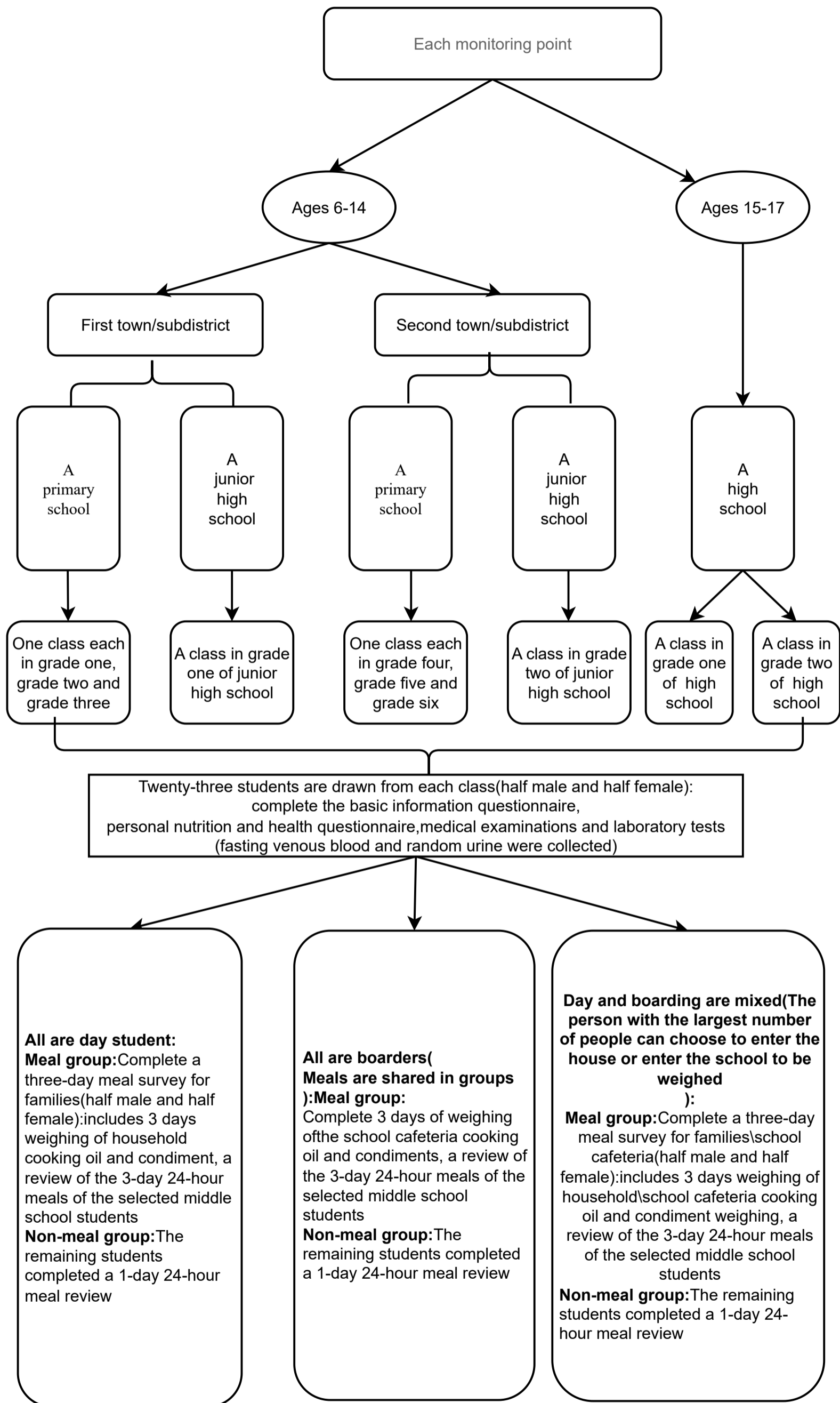

Supplement: Supplementary file 1 [file Datasheet1.pdf]
