# Supplementary material for: The relationship between secondhand smoke exposure in Chinese children and adolescents and renal function and hyperuricemia: a cross-sectional study
Source: Front Pediatr. 2026 Jun 9;14:1793355. doi: 10.3389/fped.2026.1793355 (PMC13287010; doi:10.3389/fped.2026.1793355)
Supplement: Supplementary file 2 [file Datasheet2.docx]

Appendix 2 Informed consent for this study

**Informed Consent Form for the Nutrition and Health Monitoring of Chinese Children and Lactating Mothers**

**(For Children Aged 6 - 17 and Lactating Mothers)**

Dear Parent,

We are the staff of __________. We are currently conducting a nutrition and health monitoring project for children and adolescents aged 0 - 17 and lactating mothers in China. You or your child has been randomly selected as a survey participant. Before you decide whether your child or you will participate, we would like to explain the purpose, content, potential risks, and benefits of this survey.

The nutrition and health status in the early stages of life are crucial for a person's lifelong development. Malnutrition and nutrition - related health problems caused by an unreasonable diet structure during childhood and adolescence remain severe. In order to understand the current prevalence and changing trends of the nutrition and health status and nutritional diseases of children and adolescents aged 6 - 17 and lactating mothers in China, and to provide a scientific basis for the government to formulate policies to improve the nutritional status of children aged 6 - 17 and lactating mothers, the National Health and Family Planning Commission has entrusted the Chinese Center for Disease Control and Prevention to conduct a sampling survey in 150 monitored counties (districts) across the country.

If you participate in this survey, the survey content will include:

(1) Questionnaire items such as general information, personal living habits, and health status. □

(2) Physical examinations including height, weight, waist circumference, and blood pressure. □

(3) Dietary intake over 3 days. □

(4) Collection of 6 ml of fasting venous blood to test indicators reflecting nutritional status such as whole - blood hemoglobin, vitamin A, vitamin D, serum ferritin, serum transferrin receptor, serum high - sensitivity C - reactive protein, and serum zinc; routine biochemical indicators such as blood glucose, 4 lipid items, albumin, total protein, creatinine, and uric acid. □

(5) Collection of random urine samples to test urinary sodium, iodine, urinary protein, and urinary creatinine. □

During the on - site investigation, you are required to complete your child's questionnaire and physical examination with your child at the physical examination site. It will take about 1 hour of your time. The relevant physical examination costs are covered by national financial funds. This is different from a regular physical examination. Only very few survey participants may experience symptoms such as hypoglycemia, fainting during blood drawing, or mild bruising at the blood - drawing site during the physical examination. However, please rest assured that we are equipped with relevant medications, first - aid equipment, and medical staff during the on - site investigation. In case of an emergency, we can handle it promptly.

The survey will be carried out by well - trained investigators. They will explain every question to address your concerns. We have dedicated personnel to safeguard all information that may involve your child's personal privacy and ensure strict confidentiality. When the results are released, only the survey results of the group will be reported, and no individual results will be disclosed. Through these measures, we will minimize the risk of information leakage. If you agree to participate in this survey, we will feedback the results of your child's physical measurements and hemoglobin within 2 weeks after the on - site investigation. Within 3 - 6 months after the on - site investigation, we will provide you with the test results of indicators reflecting nutritional status such as vitamin A, vitamin D, serum ferritin, serum transferrin receptor, serum high - sensitivity C - reactive protein, and serum zinc; as well as blood glucose, 4 lipid items, albumin, total protein, creatinine, and uric acid. We will also offer you further health consultations and a small gift to express our gratitude for your active participation in this survey. Of course, you have the right to refuse to participate or withdraw from the survey at any time without any penalty.

Your participation will enable us to better understand the nutrition and health status of children aged 6 - 17 and lactating mothers, thus providing support for the country to formulate relevant policies. Therefore, we sincerely hope that you can strongly support and cooperate with this survey!

Since investigators need to obtain information from you in person during the survey, it may take up some of your time. We apologize for the inconvenience. If you have any relevant health problems or suggestions, please feel free to let us know, and we will do our best to help. You can also directly contact Zhao Liyun, Wang Limin, or Zhang Jian from the Chinese Center for Disease Control and Prevention. If you have any ethical issues related to your personal rights and interests, you can contact Chen Liang from the Chinese Center for Disease Control and Prevention.

Contact Person: Zhao Liyun Contact Person: Wang Limin

Tel: 010 – 83132929 Tel: 010 - 83136482

E - mail: liyun1964@vip.sina.com E - mail: wlm65@126.com

Contact Person: Chen Liang Contact Person: Zhang Jian

Tel: 010 - 58900228 Tel: 010 - 83132560

E - mail: E - mail: zhjian6708@126.com

**Informed Consent Statement:**

I have read this informed consent form and am aware of the survey's purpose, content, risks, and benefits. All my questions have been answered. I voluntarily consent to my child's participation in this survey.

On - site investigation (including questionnaire survey, physical examination, and fingertip blood collection): Agree □ Disagree □

Name of the Parent of the Survey Participant (Signature): ____________ Date: ______ Year ______ Month ______ Day

Name of the Child/Adolescent Survey Participant (Signature): ____________ Date: ______ Year ______ Month ______ Day

I have explained the survey purpose to all survey participants and answered all their questions. As far as I understand, the survey participants are aware of the research purpose, content, risks, and benefits.

Name of the Staff Obtaining Informed Consent (Signature): ____________ Date: ______ Year ______ Month ______ Day
