# Supplementary material for: The relationship between secondhand smoke exposure in Chinese children and adolescents and renal function and hyperuricemia: a cross-sectional study
Source: Front Pediatr. 2026 Jun 9;14:1793355. doi: 10.3389/fped.2026.1793355 (PMC13287010; doi:10.3389/fped.2026.1793355)

Appendix 4 Standardized regression coefficients of the included parameters in lasso regression. (Based on the dotted lines in the upper part of Figure 1, five variables were selected for inclusion in the logistics regression model, namely Sex, age, BMI, SHS, and triglycerides.)


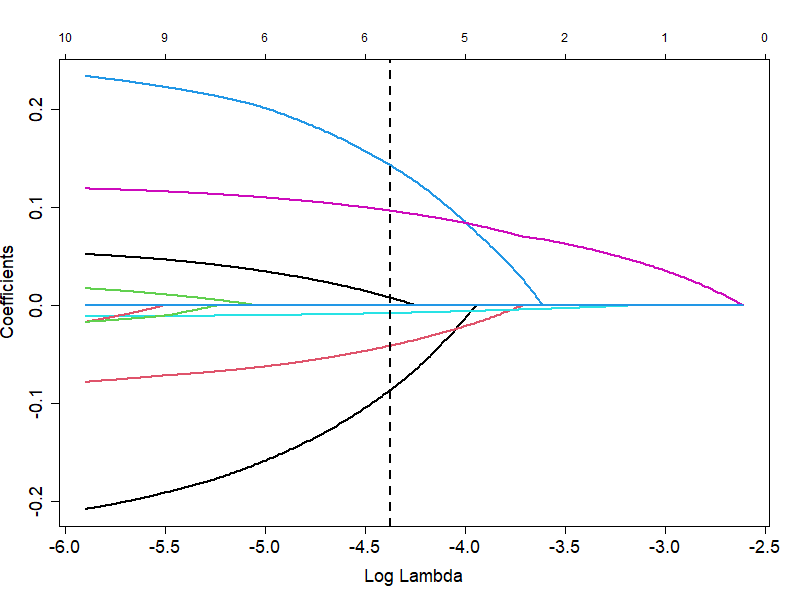

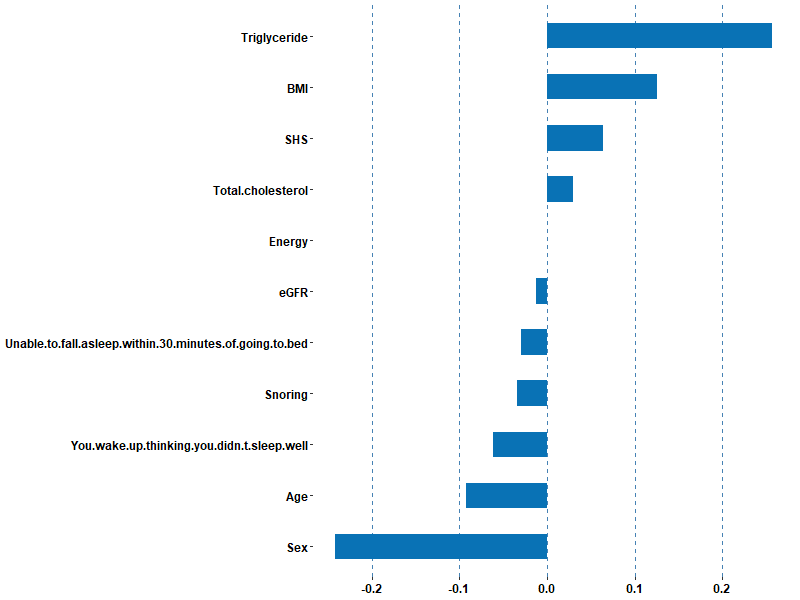

Supplement: Supplementary file 3 [file Datasheet3.docx]
